# Effects of *Melissa officinalis* L. Essential Oil in Comparison with Anaesthetics on Gill Tissue Damage, Liver Metabolism and Immune Parameters in Sea Bass (*Lateolabrax maculatus*) during Simulated Live Transport

**DOI:** 10.3390/biology11010011

**Published:** 2021-12-22

**Authors:** Qi Wang, Jun Mei, Jie Cao, Jing Xie

**Affiliations:** 1 College of Food Science and Technology, Shanghai Ocean University, Shanghai 201306, China; m190310920@st.shou.edu.cn (Q.W.); jmei@shou.edu.cn (J.M.); m190300743@st.shou.edu.cn (J.C.); 2 National Experimental Teaching Demonstration Center for Food Science and Engineering, Shanghai Ocean University, Shanghai 201306, China; 3 Shanghai Aquatic Products Processing and Storage Engineering Technology Research Center, Shanghai Ocean University, Shanghai 201306, China

**Keywords:** *Lateolabrax maculatus*, live transport, MOEO, anaesthetic, stress

## Abstract

**Simple Summary:**

The traditional method of transporting live fish in water often involves long driving durations and a high transport density. These conditions can induce stress in the fish, severely effecting their immune system and organs, such as the gills, liver and gut. In this study, *Melissa officinalis* L. essential oil (MOEO) and two kinds of common anaesthetics were applied to live fish during transport to reduce mortality and stress responses in sea bass (*Lateolabrax maculatus*). Sea bass were transported for 72 h with a transport density of 250 kg/m^3^ in different concentrations of MOEO and anaesthetics. The effect of MOEO on tissue damage, energy metabolism and on some oxidative stress and immune parameters of the animals were determined and compared with the effect of the anaesthetics. The results of this study indicated that the energy metabolism, gill and liver tissue damage and immune responses of anesthetized and sedated fish were lower than the control fish. MOEO can reduce the effects of stress and tissue damage in live fish when used as a novel sedative and anaesthetic.

**Abstract:**

In the current study, *Melissa officinalis* L. essential oil (MOEO), a novel sedative and anaesthetic, was employed in transport water to obtain a lower stress effect and higher survival rate for live marine fish. The effect of MOEO and various types of anaesthetics, administered at a low temperature on gill morphology, liver function and immunological parameters of living sea bass (*Lateolabrax maculatus*) subjected to transport stress, was evaluated to optimize the anaesthetic and sedative concentrations during live sea bass transport. Light microscopy and scanning electron microscopy of sea bass, subjected to simulated live transport for 72 h, demonstrated that the changes in the morphological characteristics of gill tissue treated with 40 mg/L MOEO (A3 group) were minimal in comparison to those observed in untreated sea bass. The results of pyruvate kinase (PK), phosphofructokinase (PFK), hexokinase (HK), hepatic glycogen (Gly), superoxide dismutase (SOD), lipid peroxides (MDA) and Caspase-3 assays indicated that the glycolysis rate, energy consumption, lipid peroxidation and hepatocyte apoptosis were the lowest in the A3 group. The values of the two immune parameters, lysozyme (LZM) and fish immunoglobulin M (IgM), indicated the strongest immunity ability in the A3 group. After 12 h recovery, sea bass treated with 30 mg/L MS-222 (B group) displayed a 100% survival rate, sea bass treated with 20 mg/L (A2 group) and 40 mg/L (A3 group) MOEO displayed a 96% survival rate, sea bass treated with 20 mg/L eugenol (C group) had a 94% survival rate, and untreated sea bass (CK group) had a 50% survival rate. Therefore, the addition MOEO to the transport water had anaesthetic and sedative effects similar to MS-222 and eugenol. The results confirmed that the addition of MOEO to the transport water could reduce tissue damage, energy metabolism, and the oxidative stress response in sea bass during transport.

## 1. Introduction

Sea bass (*Lateolabrax maculatus*) is rich in protein and is the main marine commercial fish in China. Live sea bass are sold at a high price compared to refrigerated and frozen products [1]. However, the edible value of aquatic products falls sharply during storage. One possible reason for this is that muscle tissue is highly susceptible to deterioration caused by endogenous enzymes [2,3]. Consequently, people are increasing their consumption of live aquatic products in their day-to-day lives. However, aggressive sea bass bear many sharp spines on their backs and for this reason are vulnerable to injury during transport. Sea bass experience significant stress during keep-alive transport compared to their normal conditions, and high-density closed live transport usually results in the rapid deterioration of water quality and increased fish mortality due to the high concentration of metabolites and excretory products in the water [4]. The deterioration in water quality leads to low dissolved oxygen levels and high carbon dioxide levels resulting from the fishes’ respiration, and a decrease in pH combined with an increase in ammonia levels can cause ammonia poisoning of the animals during live fish transport [5]. At the same time, liver and gill tissues are vulnerable to environmental changes [6]. A series of chain reactions occur within the organism when fish are subjected to stress. The levels of hormones such as cortisol and adrenaline tend to increase sharply during the initial stage of the stress response [7]. When live transport time is prolonged, one of two typical responses are induced in fish by stress: the fish may gradually adapt to the stressors through self-regulation, or adverse reactions or death may occur by exhaustion of the animal or damage inflicted directly by stressors. These adverse reactions may include disorders of the respiratory metabolic system and the endocrine system, abnormalities in immune functions, injuries to gill, liver and kidney tissue, tissue cell apoptosis, decreased disease resistance and other changes [8,9,10]. Fortunately, current research directed toward improving the immune ability and enhancement of the stress resistance of fish is promising [11,12,13]. Live transport of edible fish has a crucial value in international trade, and one essential prerequisite for keep-alive transport is that the transported fish remain alive and experience a low stress response [7]. Therefore, it is necessary to explore the mechanism through which the stress response that occurs during the process of live fish transport results in tissue damage and changes in immune function. Based on this information, it should be possible to develop a stress reducing program for live fish that prolongs their survival time and ensures their viability.

Anaesthetics are often used to produce unconscious fish and thereby reduce the stress sensitivity and stress responses of the animals during live transport [14]. The temperature of the water is a significant element for keep-alive transport, and the implementation of a suitable low-water temperature during transport generally reduces the respiratory metabolic rate of the fish and lessens the deterioration of water quality. Therefore, low-temperature dormancy is widely used in live fish transport, especially when combined with anaesthetics and sedatives [15,16]. Chemical anaesthetic agents include 3-aminobenzoic acid ethyl ester methanesulfonate (MS-222) and eugenol, all of which have been commonly used in the live transport of aquatic products in recent years. Anaesthesia reduces the sensitivity of fish to stressors by inhibiting the central nervous system, and the density of fish during live transport can be increased through the use of anaesthetics [17,18,19]. Existing studies have clarified that when the anaesthetics MS-222 and eugenol are used, induction time and recovery time are related to the concentration of the anaesthetic [20]. In risk assessment studies, in which the presence of anaesthetic residues was investigated, anaesthetic residues were found to be present in 10.6% of the samples, and the levels of such residues were higher in seawater fish than in freshwater fish [21]. According to one report, the use of MS-222 to anaesthetize live edible fish is permitted in the United States, as long as the animals are held for a 21-day resting period in the absence of the drug before being sold on the market [22]. However, the abovementioned rules do not exist in China, and the public has doubts and concerns about the safety of the use of anaesthetics in fish sold for human consumption. The primary reason for this is the lack of relevant laws and the fact that implementation of regulations and standards for the use of various types of anaesthetics in fisheries is imperfect in China.

*Melissa officinalis* L. is also known as American mint and wild bergamot. *Melissa officinalis* L. essential oil (MOEO) contains aldehydes, terpenes, and phenolic compounds such as citral, rosmarinic acid and flavonoids [23,24]. *Melissa officinalis* L. extract, an anti-anxiety drug and a mild tranquilizer, plays a key role in calming and relieving the symptoms of disease. Its mechanism of action involves rosmarinic acid (RA), a compound that inhibits γ-aminobutyric acid (GABA) transaminase activity and slows the degradation of GABA, thereby maintaining the stability of GABA concentrations in living organisms [25]. Cortisol and other hormones of live fish could be reduced obviously by using citral of MOEO as the novel anaesthetic and sedative during live fish handling process [26], and the phenolic compounds and flavonoids present in *Melissa officinalis* L. have high antioxidant activity without cytotoxicity and have been used as natural antiseptics and lipid-soluble antioxidants [27], so *Melissa officinalis* L. is safe for humans. Some studies have reported that MOEO has multiple functions, including antibacterial, antiviral, anti-oxidation and free radical scavenging activity and that it improves blood glucose levels [6,28]. Jeusette et al. [29] found that addition of *Melissa officinalis* L. to the diet of cats was highly efficient in reducing the animals’ stress response. It is regrettable that there has been no practical application of *Melissa officinalis* L. in the transport of live fish. Essential oils have a wide range of uses as natural plant extracts [30]. Khumpirapang et al. [31] developed Alpinia galanga oil (AGO) as a novel anaesthetic for fish; in that study, MS-222 was used, and its effects on the blood physiology and biochemistry of koi carp were compared with the effects of anaesthetics. The study indicated that AGO is a promising natural source of an alternative fish anaesthetic. Recently, Rodrigues, et al. [32] processed *Nectandra grandiflora* essential oil to a nanoemulsion (NEN) product as a novel anaesthetic and sedative for fish. Their study showed that the resulting essential oil nanoemulsions contain more than 95% of the original ingredients and that the major compounds remained stable for up to 60 days. They exposed Nile tilapia to NEN at a concentration of 30 mg/L for keep-alive transport, and the survival rate was 100% after 72 h of transport. Of course, the results of this study can also be applied to the emulsification of other types of essential oils, and this will help us explore and identify additional anaesthetics and sedative essential oils that may be beneficial for use in live fish transport. In this study, a natural plant extract of MOEO was used as a novel sedative and anaesthetic during the live transport of edible fish. The fish were transported under low temperature conditions combined with treatment with *Melissa officinalis* L. and various types of anaesthetics. The objective of the research was to improve the animals’ immune ability and increase their survival rate by decreasing the stress effects and the resulting tissue damage. The results of this study will contribute to exploiting a novel and effective anaesthetic, enhance animal welfare, and increase the live transport efficiency and survival rate of marine fish.

## 2. Materials and Methods

### 2.1. Preparation of Sea Bass

Sea bass (500 ± 120 g, 39 ± 1 cm) were purchased from the local market in Luchao Port town (Shanghai, China) and transported to the laboratory in a live fish transport box. All of the fish were healthy, breathing regularly, and active, and then were temporarily cultured for 36 h without feeding [33]. The parameters of the culture were as follows: the density of culture was 20 kg/m^3^, the water temperature was 20~22 °C, the salinity was 16‰, the dissolved oxygen was 4~6 mg/L, and the pH was 7.5~8.5. After temporary cultivation, the temperature was decreased from 20~22 °C to 12 °C at a rate of 3 °C/h. These temperatures are within the natural tolerance of this species (33.6~2.7 °C) [34,35,36].

### 2.2. Experimental Design

#### 2.2.1. Pre-Experiment: Determination of the Use of MOEO and Anaesthetic

The other parameters of the transport water were the same as those of the temporary culture, and the levels of ammonia, nitrite and nitrate in the water were approximately equalled to 0 mg/L before transport. Next, the sea bass were divided into groups and subjected to simulated transport for 72 h, the transport density was 250 kg/m^3^. MOEO was purchased from Gaodao essential oil trading company (Chongqing, China), the main compounds were citral (44.9%), geraniol (21.1%), citionella (15.4%), citronellol (6.3%), rosmarinic acid (4.1%) and D – limonene (2.3%) etc. Five groups were treated with MOEO (10 mg/L, 20 mg/L, 40 mg/L, 60 mg/L, and 80 mg/L), five groups were treated with MS-222 (10 mg/L, 20 mg/L, 30 mg/L, 40 mg/L, and 50 mg/L). Because MS-222 was weakly acidic, the pH value range of the water after adding MS-222 was 7.0~8.0. Five groups were treated with eugenol (10 mg/L, 15 mg/L, 20 mg/L, 25 mg/L, and 30 mg/L), the eugenol solution was prepared in a mixture of alcohol and Tween-80 (200:1, *v*/*v*). Samples from sea bass kept in water to which none of these agents was added were used as controls (CK). The number of animals used in each group was 20 and a total of 320 animals were used in the pre-experiment. This project was ethically reviewed, and a licence or permit was given (SHOU-DW-2021-067) and the number of that ethics approval was 400. The optimum concentration of each agent was determined based on the results of the pre-experiment and applied for 2.2.2. The survival rate of the animals in each group was determined [37,38].

(1) $$\mathrm{Survival}\;\mathrm{rate}=\frac{\mathrm{Number}\;\mathrm{of}\;\mathrm{surviving}\;\mathrm{fish}}{\mathrm{Number}\;\mathrm{of}\;\mathrm{fish}\;\mathrm{in}\;\mathrm{sample}}\times 100\%\;$$

#### 2.2.2. Experiment: Simulation of Live Transport of Sea Bass

The MOEO solution was prepared in a mixture of alcohol and Tween-80 (200:1, *v*/*v*); the concentrations of MOEO in the culture water were 10, 20 and 40 mg/L. The concentration of MS-222 in the culture water was 30 mg/L, and the concentration of eugenol was 20 mg/L. The control (CK) group was similar to that of the pre-experiment. After temporary cultivation, the temperature was decreased from 20~22 °C to 12 °C at a rate of 3 °C/h, which cannot exclude that cooling down a warm water fish species so quickly has an influence on the physiology of the fish. However, all fish were equally treated in this respect. The number of animals used in each group was 30 and a total of 180 animals were used in the experiment. The sea bass were stocked and transported in live fish transport boxes containing identical volumes of cold water (12 °C) that had been supplemented with various concentrations of MOEO and/or anaesthetics in advance [39]. The duration of the simulated transport was 72 h, the transport density was 250 kg/m^3^, the temperature of transport was 12 °C and the water quality during simulated transport was the same as the water quality in the temporary cultures. The simulated transport consisted of transport for 1 h on a B-level of unpaved roads (80 km/h) followed by 4 h of transport on an A-level of tarmac roads (100 km/h) and then 1 h of transport on a B-level road (80 km/h); this cycle was repeated 12 times. Three sea bass were randomly selected for sampling when 2, 4, 6, 8, 10 and 12 transport cycles had been completed, and sampling without changing the water. The dead fish were removed from the tanks during the transport of live fish. After 72 h of simulated transport, the sea bass were recovered and temporarily cultured in water at room temperature [40].

### 2.3. Pre-Treatment of Sample and Determination of Indicators

The sea bass were stunned by placing them in iced water for 15 min, but without coming into contact with the ice, and then sacrificed in accordance with the principles and guidelines established by the Animal Care and Use Committee of Shanghai Ocean University (SHOU-DW-2021–067). Blood was taken from the tail vein without the use of anticoagulant. The blood was stored at 4 °C for 2 h and then centrifuged at 10,614 g for 5 min at 4 °C; the resulting supernatant (serum) was collected. The serum was stored at −80 °C until use. Note: The serum used to measure immune indices should not be used after repeated freezing and thawing. The upper part of the gill filament was removed from the second gill arch, and the gill tissues were immersed in 4% formaldehyde solution for 24 h and then in 2.5% glutaraldehyde solution for 24 h [10]. The gill tissues were then observed by light microscopy and scanning electron microscopy. Finally, the animal was placed on an ice tray, its liver was dissected, and relevant indices of liver tissue and immunity were determined.

#### 2.3.1. Light Microscopy of Gill Tissue

The gill filaments were fixed with 4% paraformaldehyde solution in phosphate-buffered saline (PBS, pH = 7.4) for 24 h, dehydrated in graded ethanol, cleared in xylene, embedded in paraffin and cut into 4-μm slices using a microtome (Leica, RM2135, Nussloch, Germany). The gill slices were stained with haematoxylin-eosin (Sigma–Aldrich), rehydrated by passage through a graded alcohol series, treated with xylene, and sealed with neutral gum according to the manufacturer’s instructions. Light microscopy was performed using an optical microscope (Olympus BX-43, JESCO, Tokyo, Japan).

#### 2.3.2. Scanning Electron Microscopy of Gill Tissue

Fixed samples were washed with 0.1 mol/L PBS (pH = 7.4) three times and dehydrated in a graded ethanol series. Isoamyl acetate was used to replace the ethanol, and the samples were coated with a gold-plated conductive coating before observation. The surface of the gill tissue samples was observed by thermal field emission scanning electron microscopy (SU5000, Hitachi, Hitachi High-tech Corporation, Tokyo, Japan) at an accelerating voltage of 5 kV.

#### 2.3.3. Determination of Liver Injury Indices

Pyruvate kinase (PK) activity was determined by Baldissera et al. [41]; Phosphofructokinase (PFK), hexokinase (HK), hepatic glycogen (Gly), lipid peroxides (MDA) and caspase-3 activities in the liver and superoxide dismutase (SOD) activities in the serum were measured spectrophotometrically using commercial kits (Jiancheng Bioengineering Institute, Nanjing, China).

#### 2.3.4. Determination of Blood Immune Indices

The lysozyme (LZM) activity (μg mL-1) of serum was analysed using a turbidimetric assay. Commercial fish ELISA kits for immunoglobulin M (IgM) were supplied by Jiancheng Bioengineering Institute (Nanjing, China).

## 3. Statistical Analysis

The two-way ANOVA-Duncan test program in SPSS 21.0 software was used for multiple comparisons; Using Levene test check the homogeneity of the samples before applying Duncan. The results are expressed as mean ± SD. Origin software was used to create graphs.

## 4. Results and Discussion

### 4.1. Survival Rates of Sea Bass

Live sea bass were transported while undergoing treatment with MOEO at 10, 20, 40, 60, or 80 mg/L, MS-222 at 10, 20, 30, 40, or 50 mg/L, eugenol at 10, 15, 20, 25, or 30 mg/L, or none of these agents (the CK group). The survival rates observed during long-distance simulated live transport were presented in Table 1. The survival rates after 12 h of recovery of the animals treated with 10, 20 and 40 mg/L MOEO were all above 80%, and the survival rates of the groups treated with 20 mg/L and 40 mg/L MOEO were the highest (96%). After recovery for 12 h, the survival rate of the group treated with 30 mg/L MS-222 was 100%, and that of the group treated with 20 mg/L eugenol was 94%. However, the survival rate of the CK group was 50%. The group treated with MS-222 had the highest survival rate observed in this study, and the survival rates of the animals in the 20 mg/L and 40 mg/L MOEO groups after live transport were intermediate between that of the MS-222 group and that of the eugenol group. We conclude that MOEO can be recommended for future research on live fish transport.

### 4.2. Light Microscopy of Gill Tissue

**Figure 1:**
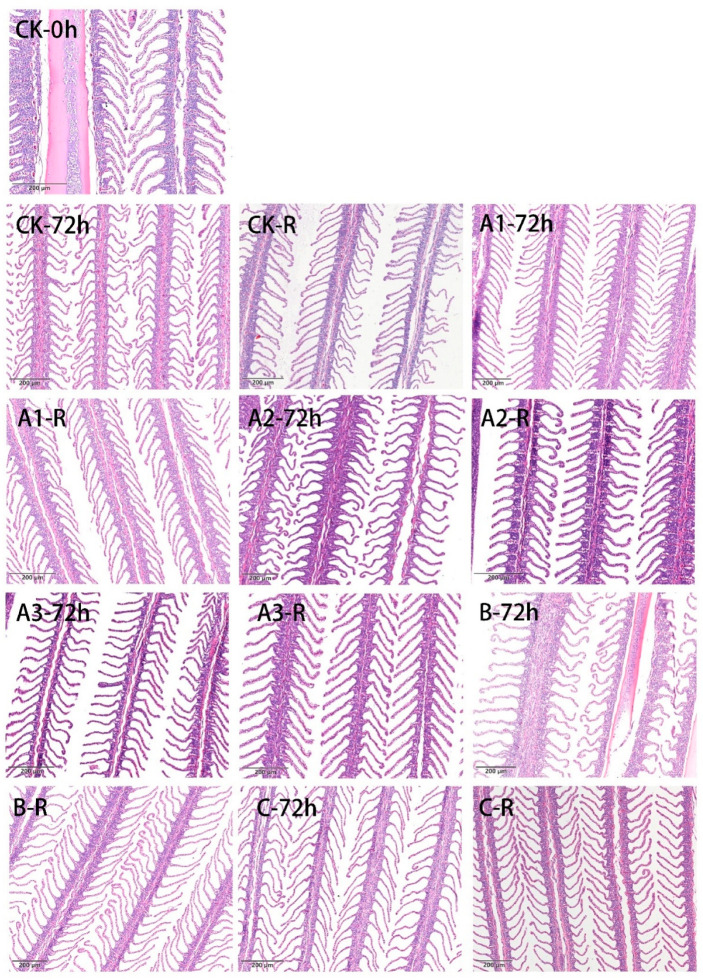
Representative H&E stained sections micrographs of the effect in different treatments on the gill structure of sea bass during keep-alive transportation. A1: 10 mg/L MOEO, A2: 20 mg/L MOEO, A3: 40 mg/L MOEO, B: 30 mg/L MS-222, C:20 mg/L eugenol, CK: no sedative of anaesthetic addition. After 72 h exposure or 12 h after recovery (R), other groups were also represented in this way.

The fish gill, a complex physiological organ, is the main site of gas exchange in fish; it executes diverse physiological functions such as ion and acid-base regulation and nitrogenous waste excretion [42,43]. Histological evaluation of H & E stained gill tissue under a light microscope was conducted after long-distance simulated live transport for 72 h and recovery for 12 h. As shown in Figure 1, the gill lamellae of the sea bass in the CK group were wide and regular, without deficiencies, prior to transport (Figure 1, CK-0 h). However, the gill filaments began to shrink, collapse, bend and fold to varying degrees with increasing transport time. Literature observations have shown that the respiratory surface of the gills of fish may increase in response to a reduction in the oxygen content of the water [44], similar to the results reported by Nie et al. [10]. After 72 h of transport, the gill filaments in the A1–72 h, A2–72 h, and A3–72 h samples exhibited slight shrinkage and deformation compared with the other samples; after recovery for 12 h, the A2 and A3 samples gradually began to resemble the pre-transport sample (0 h). The gill filaments of the CK samples presented an inferior condition, and the gill lamella of the animals in these samples were seriously disordered and appeared shrunken and bent after 72 h of transport. The gill filaments of these animals also exhibited brittle fractures after recovery for 12 h. The rosemary and ketones in MOEO played a significant role in alleviating oxidative stress and enhanced the effect of calmness and sedation. The gill filaments in the B and C groups presented abnormal curvature and contraction after 72 h of live transport (Figure 1, B-72 h, C-72 h), although Groups B (Figure 1, B-R) and C (Figure 1, C-R) showed a gradual tendency toward better gill tissue morphology; however, the distance between the gill lamellae in these animals was wide, and there were still differences from the CK-0 h animals after the 12-h recovery period (Figure 1, B-R, C-R). Sea bass may suffer from irreversible damage during simulated transport or, alternatively, may require a longer time to return to their original state. Mata et al. [45] reported that the gill filaments of fish under stress showed disorganization of the epithelium, with significant spaces between the filaments. The brittle filaments and the increased acidity caused by anaerobic breathing during simulated transport could produce contraction and breakage of the gill lamellae. Changes in gill tissue such as cell proliferation and gill bending represent a defence mechanism through which sea bass attempt to maintain normal life activities at low temperatures during simulated transport. These changes in tissues and organs were caused by weakening respiratory function and decreasing immune metabolism. Furthermore, ammonia, the main product of nitrogen metabolism, typically causes stress-induced alterations in fish gills if ammonia accumulates to a level that exceeds the threshold tolerated by fish [46]. The responses of the gills and variations in their condition are easily identified and provide valuable detail that reflects the health conditions of fish [47].

### 4.3. Scanning Electron Microscopy of Gill Tissue

**Figure 2:**
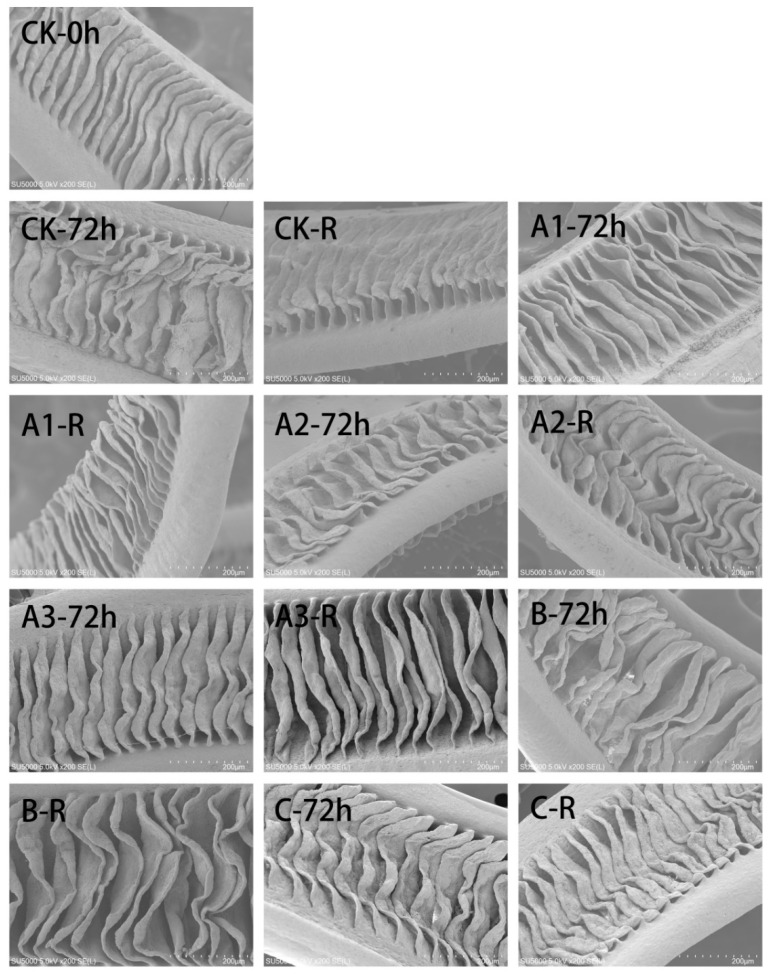
Representative scanning electron micrographs of gill structure. The groups represented by different letters and numbers are the same as in Figure 1.

Photomicrographs of gill samples examined by scanning electron microscopy (SEM) are shown in Figure 2. After simulated transport for 72 h, the MOEO-treated sea bass did not suffer obvious alterations in gill structure compared with the 0-h samples (Figure 2, A1–72 h, A2–72 h, A3–72 h, CK-0 h). However, the small gill pieces displayed serious shrinkage and deformation, the reason behind this result could be that the filaments within the gills in the CK samples appeared hyperplastic to obtain more oxygen for organisms after simulated transport (Figure 2, CK-72 h) [10]. The sea bass in the CK group displayed a strong stress effect and sensitivity to the environment and produced high respiratory metabolism in comparison with the other groups during simulated transport. The gills of the fish in the MS-222 group showed disordered patterns (Figure 2, B-72 h). The SEM results were consistent with the light microscopy results. The gills of the fish in the eugenol group (Figure 2, C-72 h) displayed mild shrinkage of the surface area on both sides of the surface folds, and the slight deformation and contraction of the gill tissue indicated that the injury was slight. The reason for this is that the anaesthetics did not protect the animals against oxidative stress, and the anaesthetic is gradually metabolized by the fish as the live transport time is extended. The increase in respiratory metabolic rate causes an accumulation of excretory products, aggravating the effects of stress and oxidative damage and resulting in disordered gill tissue in the sea bass [48]. The gill tissue of the animals in each group recovered gradually through self-regulation after a period of 12 h (Figure 2, CK-R, A1-R, A2-R, A3-R, B-R, C-R). At this time, the gill lamellae of the animals in the A3 group were orderly and abundant. Thus, it was confirmed that MOEO has calming and soothing effects that efficiently reduce gill tissue damage and stress responses during live fish transport.

### 4.4. Determination of Liver Injury Indices

#### 4.4.1. Determination of Glycolytic Pathway Activity

**Figure 3:**
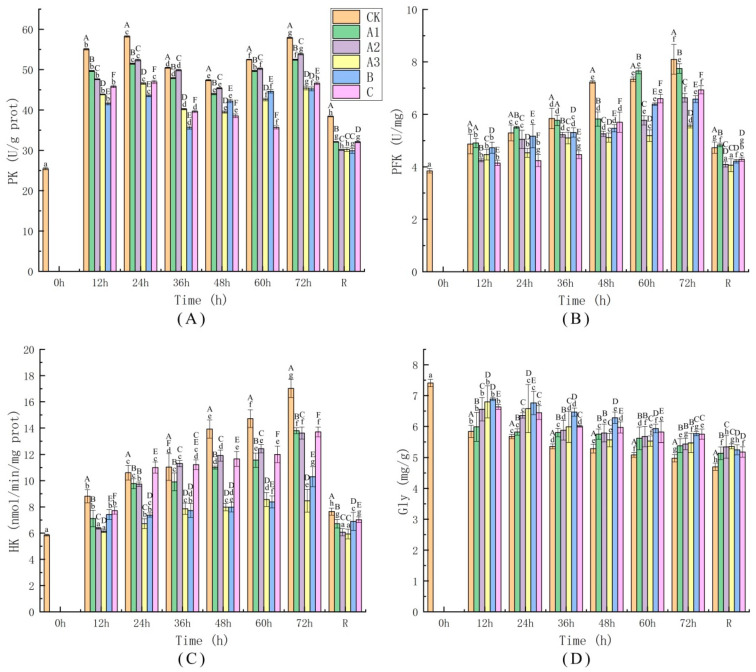
The activity changes of Pyruvate kinase (PK; (**A**)), Phosphofructokinase (PFK; (**B**)) and hexokinase (HK; (**C**)), concentrations of hepatic glycogen (Gly; (**D**)). Sea bass (*Lateolabrax maculatus*) were sampled at different time intervals in all six groups. CK: no sedative of anaesthetic addition, A1: 10 mg/L MOEO, A2: 20 mg/L MOEO, A3: 40 mg/L MOEO, B: 30 mg/L MS-222, C: 20 mg/L eugenol. After 0, 12, 24, 36, 48, 60, and 72 h exposure or 12 h after recovery (R). Vertical bars indicate standard deviation; Means with different lowercase letters indicate a significant difference between time intervals within each group (*p* ≤ 0.05); While different capital letters indicate significant differences between groups in each time interval (*p* ≤ 0.05).

The liver, as an intermediary agent associated with various organs and tissues, is a crucial metabolic organ that regulates energy metabolism [49]. Being subjected to stress for a long time can result in lipid peroxidation in liver tissue, and these consequences can compromise energy metabolism connected with the phosphor transfer network because the enzymes belonging to the phosphor transfer network are susceptible to oxidative stress [50]. Changes in the activities of enzymes related to the liver glycolysis pathway of sea bass during live transport are shown in Figure 3. The results show that PK activity displayed an increasing trend (Figure 3A). PK activity showed a tendency to increase at 60 h of transport in all treated samples; at this time, the organism needed much energy to resist changes in the environment because the water quality was seriously reduced during this period. At 12 h after recovery, PK activity in all groups showed a significant downward trend, with no significant differences among the A2, A3, and B treatment groups (*p* ≤ 0.05). According to the literature, the enzyme activity in the body increases or decreases, and the PK activity in different species of fish responds differently when oxidative stress occurs in the liver [51]. For example, Fudge, et al. [52] found that PK activity in tuna decreased with increasing temperature, while Cordiner et al. [2] reported an elevated trend of PK activity in rainbow trout with changes in temperature.

PFK activity showed a gradually increasing trend (Figure 3B); this result is similar to that of Trenzado et al. [53], who proposed that PFK levels in rainbow trout increase during stress. The CK and A1 groups presented high PFK activity in comparison with the A2, A3 and B groups, and the increase was slowest in the A3 group. The PFK activity in Group A3 was lower than that of any of the other groups at 72 h of keep-alive simulated transport, but after 12 h of recovery, the PFK activity was not significantly different from that observed in the CK-0 h sample. These results indicate that the addition of 40 mg/L MOEO (A3) to the transport water can effectively soothe transported sea bass and reduce the rate of glycolysis.

Figure 3C shows that the HK activity of the fish increased gradually with increasing live transport time. This is similar to previous research results; the present study demonstrated that cold conditions induce an increasing trend in the HK activity of fish [54,55,56]. PK activity increased at the highest rate in the CK group, indicating that the stress response of the sea bass in this group was the strongest and that the rate of glycolysis was accelerated to resist the stress response. The activity of PK in the A3 and B groups showed the slowest increase, and all of the treatment groups presented a significant decreasing trend in PK activity at 12 h of recovery. This can be interpreted as indicating that the fish did not require much energy during this period. Furthermore, there was a large amount of glucose-6-phosphate in the animals’ bodies.

The liver glycogen of the fish presented a slowly declining trend; among the groups, the CK group displayed the most significant decrease in liver glycogen (Figure 3D). This implies that the energy consumption of the CK group was the largest. The changes in liver glycogen levels that occurred during the entire live transport process matched the observed changes in glycolysis rate. In general, glycolysis was lowest in the A3 group, and the energy consumption of this group was the lowest. The results of this study show that exposure to 40 mg/L MOEO reduces energy metabolism in sea bass.

#### 4.4.2. Determination of Oxidative Stress Injury Indices

**Figure 4:**
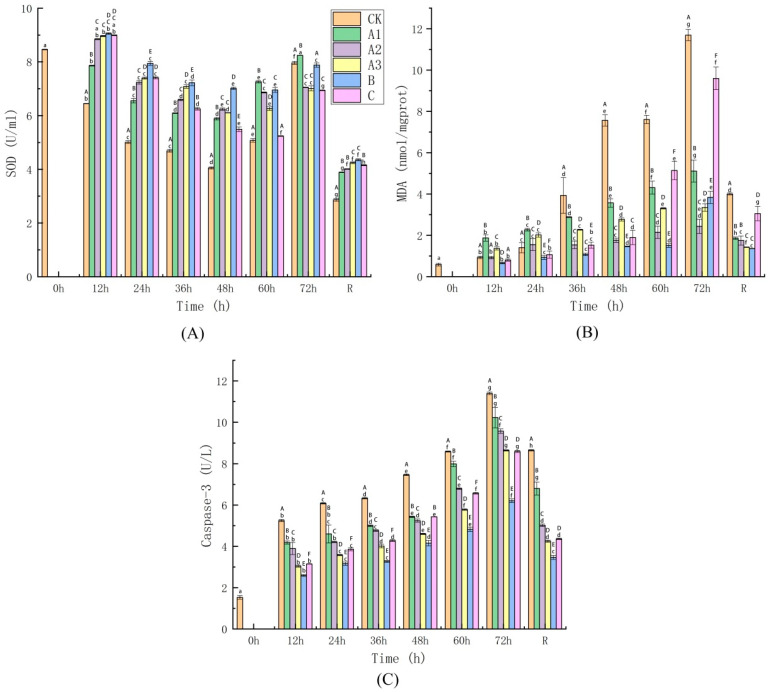
The concentrations changes of superoxide dismutase (SOD; (**A**)), lipid peroxides (MDA; (**B**)) and Caspase-3 (**C**). The groups represented by different letters and numbers are the same as in Figure 3.

As shown in Figure 4A, the SOD level first increased and then decreased during the process of continuous transport. SOD levels can indirectly reflect the capability of the body to scavenge oxygen free radicals [57]. The SOD activity in the animals’ bodies increased, but the CK group showed a decreasing trend after simulated transport for 72 h (Figure 4B). The reason for this decrease in SOD after simulated transport may be related to the response of the organisms to oxidizing agents or to the absence of anaesthetic and sedative agents to the transport water [30]. The sea bass in the CK group had higher SOD activity than the other groups. This suggests that they were sensitive to changes in the environment, and this produced a strong antioxidant reaction. There was a decreasing trend in SOD activity, which indicated that the ability to recognize free radicals was decreased. The SOD level of the A1 group was significantly higher than that of the CK group during simulated transport, and the A2 and MS-222 groups displayed the smallest and most stable variations. However, the SOD level of the CK group was significantly lower (*p* ≤ 0.05) than that of the other groups after recovery for 12 h. In contrast, the A3 group and the B group had the highest viability and displayed no significant differences in SOD levels during recovery.

Under normal circumstances, trace amounts of lipid peroxides present in fish can be removed with time by normal tissue activity [58]. Nevertheless, fish could produce excess oxygen free radicals under live transport conditions. These free radicals cause peroxidation of lipids, proteins, nucleic acids and other biological macromolecules. Peroxide-induced lipid peroxidation damage in the body is a severe type of damage, and it can cause extensive damage to liver cells, such as disruption of cellular structure and of the function of the organ [59]. Figure 4B shows that the MDA level increased sharply as the simulated transport time increased. The CK group displayed a significant difference from the other groups in its MDA level; MDA reached its peak value of 11.69 nmol/mg prot after 72 h of simulated transport. After that, the MDA level of each group decreased slightly at 12 h of recovery. Groups A3 and B reached the lowest values (1.42 nmol/mg prot and 1.75 nmol/mg prot, respectively), and there was no significant difference between them. The MDA level of the CK group was high (*p* ≤ 0.05) in comparison with that of the other groups at 12 h after simulated transport (Figure 4B). Furthermore, it appears that the effect of MOEO on MDA levels in sea bass was negatively dose-dependent; these results correspond to the trends in the changes in SOD activity (Figure 4A). Wu et al. reported that root extract of glycine efficiently mitigated stress during simulated transport of orange-spotted grouper (*Epinephelus codes*) [60]. In the current study, the addition of MOEO to the water during live sea bass transport had an obvious effect on reducing lipid peroxidation and on the production of oxygen radicals; the effective dose of MOEO ranged from 20 mg/L to 40 mg/L.

Among the physiological regulatory mechanisms of teleost fishes, apoptosis is the principal mechanism of cell death immediately after wounding [61]. In healthy cells, caspase molecules exist as pro-caspases; when caspases undergo proteolytic activation during apoptosis, caspase substrate cleavage occurs, and cells receive signals that lead to injury and death. These caspases belong to the intracellular cysteine protease family that cleaves target substrates at sites adjacent to aspartic acid residues [62]. Figure 4B shows that Caspase-3 activity increased continuously with time in every group from 12 h to 72 h of transport but that it had decreased by 12 h of recovery. The Caspase-3 level of the B group was the lowest at every time point sampled, while that of the CK group was the highest, during the process of live sea bass transport. Caspase-3 activity displayed a decreasing trend with increasing MOEO concentration, indicating that apoptosis was reduced under these conditions. Moreover, Caspase-3 activity was lowest in the B group, with no significant difference between the A3 group and the C group at 72 h or 12 h after recovery. MOEO was added to the transport water to alleviate stress injury to sea bass, and a concentration of 40 mg/L showed the best effect. The anaesthetic efficacy of MOEO is similar to that of commercial anaesthetics; however, MOEO has a better antioxidant effect than does MS-222 or eugenol.

Most importantly, the levels of MDA and Caspase-3 were the lowest in Groups A3 and B, and the results for these groups showed the best antioxidant activity; this indicates that the antioxidative constituents and flavonoids present in MOEO were effective in protecting the livers of the animals against oxidative damage during transport. Therefore, the treatment conditions used in Groups A3 and B are effective at relieving oxidative stress in the livers of sea bass during live transport.

### 4.5. Determination of Immune Indices

**Figure 5:**
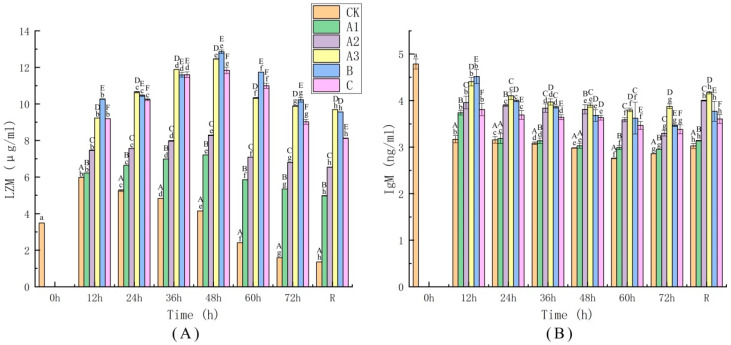
The activity of lysozyme (LZM; (**A**)) and concentrations changes of immunoglobulin M (IgM; (**B**)). The groups represented by different letters and numbers are the same as in Figure 3.

The reactions of the body to stress have long been known to influence the immune response of organisms through homeostatic regulatory mechanisms that can affect many body systems [63]. A theory of immunity has been proposed in which immunosuppression is hypothesized to be one of the mechanisms through which autoimmune damage is mitigated during exposure to stress [64]. The changes in LZM activity in the treatment groups during live transport are shown in Figure 5A. LZM activity increased at the initial stage of live transport in all groups; in the CK group, it then decreased, and after 12 h it was significantly lower than in the other treated groups (*p* ≤ 0.05). In the other groups, LZM activity displayed an upward trend until it reached a peak level of 12.86 μg/mL at 48 h of transport. LZM activity is frequently elevated during acute stress responses, as shown in recent studies of stress in Nile tilapia (*Oreochromis niloticus*) [65] and rainbow trout [66]. In the current study, LZM activity in all groups showed a decreasing trend at 60 h of simulated transport; in the CK group, it was significantly lower than in the other groups after 12 h of recovery (*p* ≤ 0.05), indicating that the immune response of the organism had been reduced. However, the A3 and B groups still had the highest levels of LZM activity and did not differ significantly from each other. This study demonstrates that addition of MS-222 and MOEO to the transport water used in live transport is effective in suppressing the decrease in immune ability that otherwise occurs in sea bass during transport. The anesthetized and sedated fish were less sensitive to the external environment and suffered less stress damage according to the above mentioned, which suggests that the immune ability of the anaesthetized fish was higher than that of the CK group during transport [67]. LZM activity of all groups increased first and then decreased during simulated transport, and the LZM activity of samples in the CK group reached a peak at 12 h after transport. This suggests that the LZM activity of samples in other treatment groups reached a peak at 48 h after transport. The LZM activity in sea bass was activated in the initial stage of transport to resist the impact of environmental stress factors on the body. After 12 h of recovery, lysozyme activity in the CK group was lower than that in the group sampled before transportation (0 h), suggesting that transport affected the immune of sea bass and the animals needed some time to recover.

Under the circumstances employed in live transport, fish are exposed to a variety of stresses, and this can lead to increased susceptibility to certain diseases. IgM is a crucial index for evaluating changes in the immune response of fish [68]. As shown in Figure 5, the IgM level (Figure 5B) was opposite to the change trend of LZM activity (Figure 5A). The IgM level of sea bass showed a downward trend with the extension of transport time, and it presented an upward trend at 12 h of recovery. Groups A2, A3, B and C showed a slow downward trend in their IgM levels, the IgM level of the A3 group was the highest, and the change trend was relatively stable throughout the live transport process. These results demonstrate that the addition of MOEO to the water used in live transport can enhance the immune function and anti-infection ability of sea bass to a certain extent. The optimal concentration of MOEO for this purpose was 40 mg/L.

## 5. Conclusions

Essential oil of *Melissa officinalis* L., as a novel anaesthetic for live fish, is less stressful and has a stronger anti-oxidative ability than the CK group during transportation. To keep the fish at a low stress level and maintain a high survival rate during live fish transport, it is essential to understand the physiological, metabolic and immune regulatory mechanisms that occur during stress resulting from live transport. Under the conditions observed for simulated transport, EOMO was safe and effective in inducing sedation and anaesthesia, being a promising option for the development of a new sedative and anaesthetic drug for use in fish. Compared with the CK group, the energy consumption level of the sedated and anaesthetized fish was lower, and the damage to tissues and organs was smaller. Adding MOEO to alleviate gill and liver tissue damage and the stress reaction of fish during live transport had an effect similar to that of the addition of anaesthetics such as MS-222. Moreover, MOEO proved to be better for the protection of gill tissue and improvement of antioxidant ability than anaesthetics such as MS-222. Therefore, MOEO can reduce the stress effect of fish when it is used as a sedative and anaesthetic in live transport; The addition of 40 mg/L MOEO to the transport water effectively slowed the damage to gill tissue and alleviated energy metabolism for the sea bass during simulated live transport. Further exploration of and research on the mechanism of action of MOEO and how it regulates fish physiology are needed. Assessment of the safety of the use of chemical anaesthetics during live fish transport and the levels of anaesthetic residues after transport need further attention.

## Figures and Tables

**Table 1:** Determination of survival rates (%) in different treatment groups during live sea bass transport and after 12 h of recovery (R-12).

Method	Keep-Alive Transportation Time/h						
	12	24	36	48	60	72	R-12
10 mg/L MOEO	100	100	100	100	92	84	80
20 mg/L MOEO	100	100	100	100	96	96	96
40 mg/L MOEO	100	100	100	100	100	96	96
60 mg/L MOEO	100	100	90	90	80	75	65
80 mg/L MOEO	100	100	95	90	75	60	45
10 mg/L MS-222	100	100	100	100	95	80	70
20 mg/L MS-222	100	100	100	100	100	85	75
30 mg/L MS-222	100	100	100	100	100	100	100
40 mg/L MS-222	100	100	100	100	95	90	90
50 mg/L MS-222	100	95	90	90	85	80	80
10 mg/L Eugenol	100	100	100	95	90	80	75
15 mg/L Eugenol	100	100	100	100	95	80	70
20 mg/L Eugenol	100	100	100	100	100	100	94
25 mg/L Eugenol	100	100	100	100	100	90	90
30 mg/L Eugenol	100	100	100	90	90	85	80
CK	100	100	90	84	76	60	50

## Data Availability

All data, models, and code generated or used during the study appear in the submitted article.
